# Biogenic silver nanoparticles improve bioactive compounds in medicinal plant *Juniperus procera in vitro*

**DOI:** 10.3389/fpls.2022.962112

**Published:** 2022-09-26

**Authors:** Abdalrhaman M. Salih, Fahad Al-Qurainy, Salim Khan, Mohammad Nadeem, Mohamed Tarroum, Hassan O. Shaikhaldein

**Affiliations:** Department of Botany and Microbiology, College of Science, King Saud University, Riyadh, Saudi Arabia

**Keywords:** biosynthesis, AgNPs, bioactive compounds, chromatographic analysis, medicinal plant, ferruginol

## Abstract

Bioactive compounds of medicinal plants present as natural ingredients provide health benefits beyond the basic nutritional value of these products. However, the availability of bioactive compounds in the current natural sources is limited. Hence, the induction of bioactive compound production from medicinal plants through nanoparticles (NPs) might play a vital role in industrially important medicinal compounds. Therefore, this study aimed to synthesize silver nanoparticles (AgNPs) biologically and to investigate their effect on phytochemical compound production from the callus of *Juniperus procera*. AgNPs were synthesized biologically using aqueous leaf extract of *Phoenix dactylifera*, which acted as a reducing and capping agent, and silver nitrate solution. The formation of AgNPs has been confirmed through different analytical techniques such as UV-Visible spectroscopy (UV), Fourier-transform infrared spectroscopy (FTIR), dynamic light scattering (DLS), and scanning electron microscope (SEM). The impact of different concentrations (0.0, 5, 20, and 50 mg/L) of AgNPs on enzymatic and non-enzymatic antioxidants of the callus of *J. procera* was investigated. The obtained results showed a significant effect of AgNPs on biomass accumulation and non-enzymatic antioxidants (phenol, tannin, and flavonoid content). Additionally, total protein content and superoxide dismutase (SOD) activity were increased in response to AgNPs. Furthermore, bioactive compounds like gallic acid, tannic acid, coumarin, hesperidin, rutin, quercetin, and ferruginol were chromatographically separated and quantified using high-performance liquid chromatography (HPLC) with reference standards. These compounds were increased significantly in response to AgNPs treatments. We concluded that AgNPs could be a promising elicitor for improving the production of phytochemical compounds in medicinal plants. This work can serve asa good model for improving the production of bioactive compounds from medicinal plants *in vitro.* This molecular investigation should be done to understand better the metabolic mechanism leading to bioactive compound production scaling.

## Graphical Abstract

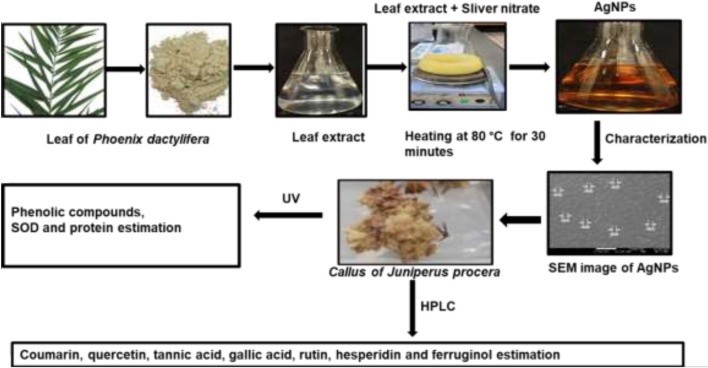

## Introduction

Nanotechnology is a new field of research that deals with the synthesis and characterization of nanoparticles (NPs) and their applications in different sectors. NPs are defined as materials with sizes of 1–100 nm or at least one dimension less than 100 nm (Hochella et al., 2008; Agnihotri et al., 2014; Frewer et al., 2014; Syedmoradi et al., 2017; Jeevanandam et al., 2018). The biosynthesis method of NPs is environmentally friendly and becoming more popular compared to chemical approaches, which are intended to reduce pollution. In addition, the advantage of the biosynthesis method lies in the availability of raw materials and their cost-effectiveness (Rauwel et al., 2015). The potential organisms used in the biosynthesis of NPs have ranged from bacterial cells to plants (Mohanpuria et al., 2008). NPs synthesis using plant extract is extremely cost-effective, simple, and safe. Hence, plants can be used as alternative materials for producing NPs on a large scale (Iravani, 2011). AgNPs have unique features which can be used in different applications, such as biosensor materials, antimicrobials, composite fibers, elicitors, cosmetic products, and electronic components (Iravani et al., 2014; Srikar et al., 2016). The major goal of inducing bioactive compounds (phenolics, flavonoids, volatile oil, terpenoids, coumarins, carotenoids, and alkaloids) in medicinal plants using NPs is to increase the quantity and therapeutic activity (Muley et al., 2009). It was suggested that nanomaterials interfere with several signaling pathways and are capable of inducing plant secondary production. The initial physico-biochemical responses of plants to nanomaterials might increase the production of reactive oxygen species (ROS), cytoplasmic Ca^2+^, and upregulation of mitogen-activated protein kinase cascades like other abiotic stresses. For example, AgNP recognition by plasma membrane-bound receptors triggered a Ca^2+^ burst and induced ROS in *Arabidopsis thaliana* (Sosan et al., 2016). Moreove*r*, levels of Ca^2+^ and proteins were found to be upregulated in the proteomic analysis of *Oryza sativa* roots treated by AgNPs (Mirzajani et al., 2014). Previous studies have provided evidence for NPs-mediated plant secondary metabolism. Besides, we have established a strong relationship between bioactive compounds production and ROS. Thus, the exposure of plants to nanomaterials induced the production of secondary metabolites (Marslin et al., 2017). In this context, phenolic compounds were increased in response to NPs treatment (Jadczak et al., 2020).

The composition of phytochemicals in plants can also be changed using biotic and abiotic elicitors; this induces a series of physiological and biochemical reactions in the plant and alters secondary metabolite production (Mulabagal and Tsay, 2004). For example, the phenolic concentrations were increased in *Arthrospira platensis* after treatment with 100 mg/L TiO_2_ NPs (García-Sánchez et al., 2015). However, the availability of phytochemical compounds in the current natural sources is limited. Thus, the elicitation of bioactive compounds in medicinal plants is needed to use them as biomolecules for human nutrition and health. AgNPs have received a great deal of attention for their distinctive physicochemical and biological properties. Therefore, it has become one of the essential nanomaterials in nanotechnology (Durán et al., 2015).

*Juniperus procera* is a vital plant with a medicinal value that can be used as an anticancer, insecticidal, and anti-microbial plant (Tumen et al., 2013; Abdel Ghany and Hakamy, 2014; Bitew, 2015). *J. procera* (Hoech stex Endl.) grows naturally in the Southern hemisphere, Saudi Arabia, and in the highlands of East Africa (Adams, 1990; Collenette, 1999). A few studies have investigated the impact of AgNPs on phytochemical compound production *in vitro*. To the best of our knowledge, there are no reports to date involving biosynthesized AgNPs’ impact on bioactive compound production from the callus of *J. procera.* Therefore, this study aimed to synthesize AgNPs biologically and investigate their impact on bioactive compound production from the callus of *J. procera*. Hence, phenolic constituents such as total phenolic content (TPC), total tannin content (TTC), and total flavonoid content (TFC) were determined using a UV-Visible spectrophotometer. Moreover, bioactive compounds such as gallic acid, tannic acid, quercetin, rutin, coumarin, and hesperidin were separated and quantified chromatographically using HPLC with reference standards.

## Materials and methods

### Chemical reagents

Methanol, acetonitrile, HPLC water, quercetin, coumarin, rutin, gallic acid, and hesperidin standards were purchased from Sigma Aldrich. The ferruginol standard was purchased from WuXi App Tec Lab Network.

### Preparation of the leaf extract

Leaves of *Phoenix dactylifera* were selected for the biosynthesis of AgNPs because of their cost-effectiveness and rich secondary metabolites (Suleiman et al., 2021). Fresh leaves of *P. dactylifera* were collected from the Botanical Garden, Department of Botany and Microbiology, College of Science, King Saud University. The leaves were rinsed thoroughly with tap water followed by doubled distilled water to remove all dust and unwanted visible particles. Then, the leaves were dried at room temperature and grounded using a blender; 5 g of leaf powder was transferred into a 250-ml beaker containing 100 ml of deionized water. The mixture was shaken for 3 h, incubated in the dark overnight at room temperature, and then filtered through 1.0- μm filter paper. The collected filtrate was used as a stabilizing and reducing agent in the synthesis of AgNPs.

### Phytochemical analysis of leaf extract

A sample from leaf extract of *P. dactylifera* used for biological synthesis of AgNPs was filtered using a 0.45 μm nylon syringe before being injected into gas chromatography-mass spectrometry (GC-MS) analysis for phytochemical screening.

### Biosynthesis of silver nanoparticles

AgNPs were synthesized biologically according to the method described by Ahmed et al. (2016) and Ashraf et al. (2016) with minor modifications. A total of 100 ml of leaf extract of *P. dactylifera* was added to 50 ml of 1 mM aqueous silver nitrate solution (2:1) (v/v) and followed by heating at 80°C for 20 min. The change preliminarily detected the formation of the AgNPs in color from light yellowish to dark brown.

### Silver nanoparticles characterization

Biogenic AgNPs were characterized using several techniques; a UV-visible spectrophotometer was performed in the range of 200–800 nm. A Fourier transmission infrared spectrometer (FTIR) was used for functional group detection. The surface charge of AgNPs was identified using dynamic light scattering (DLS), whereas surface morphology, particle size, and distribution of the silver nanostructure were measured using a scanning electron microscope (SEM) and energy-dispersive X-ray (EDX) spectroscopy.

### Media preparation

Woody Plant Media (WPM) with the addition of phytohormones, 2,4-D and BAP (2 μM), sucrose (30 g/L) was used as a source of carbon. 7 g/L of agar was added, and the pH was maintained at 5.7. Following the protocol, we recently reported (Salih et al., 2021a). Next, different concentrations of biogenic AgNPs (0.0, 5, 10, 20, and 50 mg/L) were added to the WPM before sterilizing at 121°C for 20 min. The explants were incubated in a growth chamber for 70 days for callus induction and development at 25°C ± 1, with 14- and 10-h illumination periods.

### Preparation of callus extract

The callus of *J. procera* was lyophilized before being placed in a mortar for grinding; 200 g of powdered callus was extracted using 10 mL of methanol (99.98). Then, the extraction was carried out using an Innova 44 Inc incubator for 48 h at 120 rpm, and the temperature was maintained at 28 ± 2°C. The separation of organic and aqueous phases was done by centrifugation at 5,000 rpm for 15 min. The collected supernatant was filtered through a 0.45-μm nylon syringe before usage.

### Determination of the total phenolic content

Total phenolic content (TFC) was estimated using the (Ainsworth and Gillespie, 2007) method. The reaction mixture contained 1.5 mL of deionized water, 100 μL of callus methanolic extract, and 100 μL of the Folin-Ciocalteu reagent. Next, the mixture was incubated at room temperature for 30 min and neutralized with 300 μL of sodium carbonate solution (20%, w/v). The wavelength of the resulting blue color was recorded at 765 nm using a UV–Visible spectrophotometer. The TFC was estimated using the linear equation (*y* = 0.0033x + 0.0752 with *R*^2^ = 0.9855) of the gallic acid standard.

### Estimation of total tannin content

For total tannin content (TTC) determination in callus material, the Folin–Ciocalteu method described by Rodrigues et al. (2007) was followed with slight modifications; 100 μL of the extracted callus was added to a tube containing 1.5 ml of deionized water and 100 μL of Folin–Ciocalteu phenol reagent. The mixture was shaken well and kept at room temperature for 30 min in the dark. Next, 300 μL of 35% sodium carbonate solution was added to the mixture. The wavelength of the sample and standard was measured at 700 nm. The standard was made using different concentrations (250–750 μg/mL) of tannic acid. The estimation of TTC was performed in triplicate using the following equation (*y* = 0.0054−0.0252 with *R*^2^ = 9937).

### Determination of total flavonoid content

The TFC in the callus samples was determined using the method described by Ordonez et al. (2006). A total of 0.5 mL of 2% AlCl_3_ water solution was added to 0.5 mL of extracted callus. Then, the mixture was incubated in the dark for 30 min at room temperature. The wavelength was measured at 420 nm. A standard curve was prepared using different quercetin concentrations (100–800 μg/mL). The TFC was calculated using the following equation (*Y* = 0.0042x−0.1673 with *R*^2^ = 0.9871) based on the calibration curve of quercetin.

### Determination of the total protein content

For total protein content estimation, 100 mg of plant material was grounded using liquid nitrogen and dissolved in 2 ml of phosphate buffer (pH 7.0) containing 0.5% (v/v) Triton-X 100 and 1% PVP. Then, the mixture was centrifuged at 14,000 rmp for 20 min at 4°C. The supernatant was collected, while the total protein was estimated using a NanoDrop following the method by Jogeswar et al. (2006).

### Superoxide dismutase activity estimation

Superoxide dismutase activity (SOD, EC 1.15.1.1) was determined following Marklund and Marklund’s (1974) method. The reaction mixture contained 1.5 mL of 0.1 M sodium phosphate buffer (pH 7.0), 1 mL of 6 mM pyrogallol, 0.5 mL of 6 mM ETDA, and 0.2 mL of extracted protein. The wavelength was recorded at 420 nm. SOD activity was calculated as the enzyme needed for 50% inhibition of pyrogallol oxidation.


$$\%\mathrm{Inhibition}\mathrm{of}\mathrm{pyrogallol}\mathrm{of}\mathrm{autoxidation}=\frac{\Delta Atest}{\Delta Acontril}\times 100\%$$

$$\mathrm{SOD}\mathrm{activity}\left(U/\mathrm{ml}\right)=\frac{\%Inhibitionofpyrogallolofautoxidation}{50\%}$$

### Quantification of bioactive compounds

**FIGURE 1:**
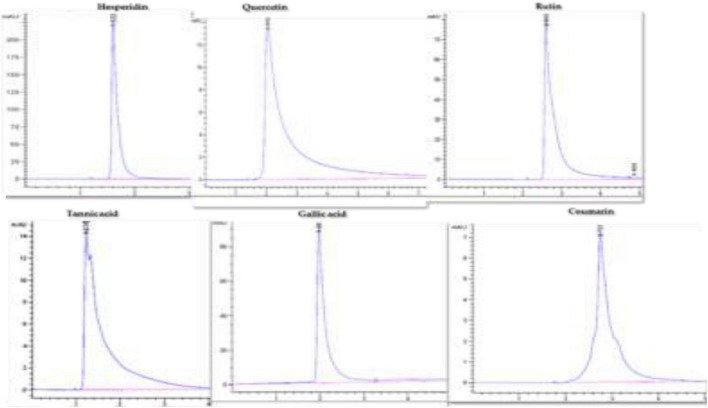
HPLC chromatograms of hesperidin, quercetin, rutin, tannic acid, gallic acid, and coumarin.

The HPLC Agilent Technologies System controlled by software (G 4226A) with the column SB-C18 (1.8 μm, 4.6 × 150 mm) was used for chromatographic analysis of targeted compounds. For separation and quantification of the bioactive compounds such as gallic acid, hesperidin, quercetin, tannic acid, coumarin, and rutin; specific standards, mobile phases, wavelengths, injection volume, and flow rate were used for each compound following Nour et al.’s (2013) and Salih et al.’s (2021b) methods. The identification of these compounds in the callus samples was possible because their retention times spiked with the specific standard of each compound under similar conditions (Figure 1). These compounds were estimated using the linear equation based on a standard curve prepared with reference standards (Table 1).

**TABLE 1:** The standards, standards concentrations, and linear equation.

Standard	Standard con. (μL)	Linear equation	*R* ^2^
Coumarin	1.5,3, 6,9	y = 140.63x−46.142	0.9982
Hesperidin	0.3,0.5,1.0	y = 1871.5x−98.923	0.9999
Rutin	0.3,0.5,1.0	y = 1540.8x−176.46	0.9998
Quercetin	0.2,0.5,1.0	y = 2017x−312.99	0.9986
Tannic acid	0.3,0.5,1.0	y = 1086.3x−117.92	0.9999
Gallic acid	0.1,0.3,1.0	y = 3874.8x−13.896	0.9998

### Ferruginol quantification

A mobile phase consisting of acetonitrile and methanol (50:60) (v/v) was used for ferruginol identification and estimation. The injected volume of the sample was 1 μl with a run time of 5 min and a 1.000 mL/min flow rate. The column temperature was maintained at 27°C. The chromatogram was measured at 220 nm. The ferruginol in the sample was identified by its retention time spiked with the ferruginol standard under similar conditions. Ferruginol was estimated using the linear equation based on a standard curve prepared with ferruginol.

### Statistical analysis

The experiment was carried out independently, at least in triplicate. The reported data presented the average of three replicates ± standard deviation (SD). Statistical analysis was performed using SPSS software, and one-way analysis of variance (ANOVA) was used to evaluate statistical significance (*P* < *0.05*).

### Legal statement

This study’s collection of plant materials complies with relevant institutional, national, and international guidelines and legislation. The seedlings of *J. procera* were collected and provided by the Botany and Microbiology Department (Garden and Herbarium Unit), College of Science, King Saud University (KSU), with the permission to collect plant materials by accepting the terms and conditions of national and international standards. The *J. procera* seedlings were identified by Prof. Ibrahim M. Arif, King Saud University, Riyadh, Saudi Arabia. A voucher specimen (# 13497) was deposited in the herbarium of the center.

## Results and discussion

This study synthesized silver nanoparticles (AgNPs) biologically using aqueous leaf extract of *P. dactylifera* and an aqueous solution of silver nitrate. Moreover, the impact of AgNPs on the callus of *J. Procera* development, physiological parameters, and bioactive compound production was investigated.

### Phytochemical screening

**FIGURE 2:**
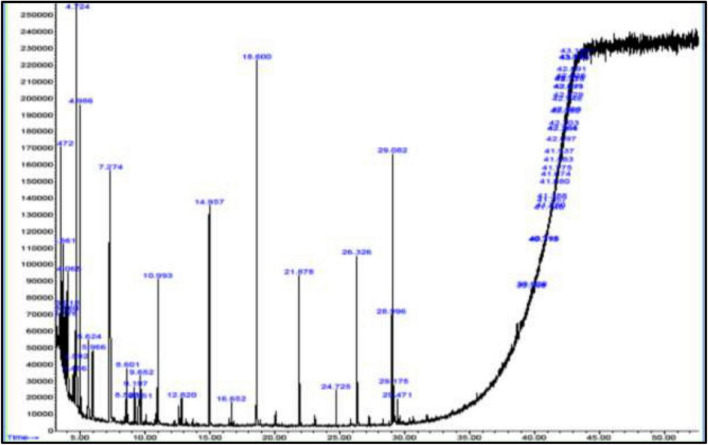
GC-MS chromatogram of leaf extracts of *P. dactylifera.*

Phytochemical screening was done to identify the presence of phytochemical compounds in leaf extract of *P. dactylifera* (Table 2) that were used as stabilizing and reducing agents in AgNP synthesis. The GC analysis of *P. dactylifera* revealed about 20 components related to phytochemical compounds Table 2 and Figure 2. These bioactive compounds can act as a scaffold, which plays the role of capping and reducing agent in the green synthesis of AgNPs (Ovais et al., 2018; Ahmad et al., 2019).

**TABLE 2:** Gas chromatographic analysis of leaf extracts of *P. dactylifera.*

Compounds	Retention time
Ethanone	3.465
Tetramethyl silicate	3.465
Benzene	4.730
Silane	4.997
Undecane	9.513
Cycloheptasiloxane	18.588
Hexadecanoic acid	26.329
Methyl 13-octadecenoate	29.082
Benzo[h]quinoline	40.204
Hexahydro pyridine	41.23
2,4,6-Cycloheptatrien-1-one	41.349
Tetrasiloxane	41.555
Phenome	41.615
Silicic acid	41.951
1,2,4-Benzenetricarboxylic acid	41.994
Hexahydropyridine	42.329
Phenoxy	42.441

### Biosynthesis and characterization of silver nanoparticles

The AgNPs used in this research were synthesized biologically using aqueous leaf extracts of *P. dactylifera* as a reducing and capping agent and silver nitrate solution. For the biosynthesis of AgNPs, 100 ml of leaf extracts were added to 50 mL of 1 mM AgNO_3_ solution (1:2) (v/v) and incubated at 80°C until the color of the mixture changed from light yellowish to dark brown. The color change is due to the excitation of surface plasmon vibration in the AgNPs. The change in color of the reaction mixture indicates the reduction of Ag + to Ag° in the AgNO_3_ solution, which confirms Ag ion reduction and the formation of AgNPs (Chandran et al., 2006; Khalil et al., 2014). Moreover, it is worth mentioning that the excitation of surface plasmon in silver causes color change in the solution (Kumar et al., 2013; Khalil et al., 2014). According to Banerjee et al. (2014), this is the first sign and notable indication of AgNP formation. Furthermore, the formation of AgNPs was confirmed by several characterization techniques (UV, SEM, DLS, and FTIR) to ascertain the morphology, shape, size, surface charge, and functionalization of NPs.

**FIGURE 3:**
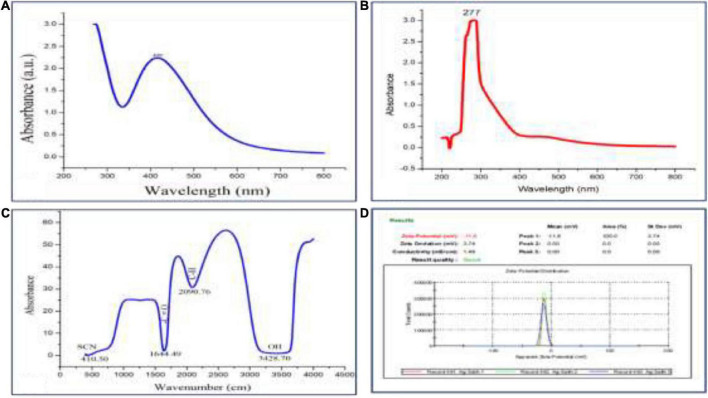
**(A)** UV–Visible absorption spectrum of biogenic AgNPs, **(B)** UV spectrum of plant extract, **(C)** FTIR pattern of AgNPs, and **(D)** Zeta potential of AgNPs.

**FIGURE 4:**
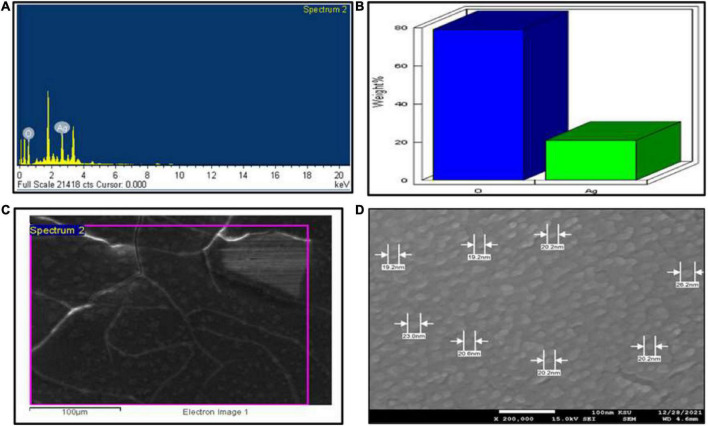
SEM investigation. **(A)** EDX spectrum of biogenic AgNPs, **(B)** quantitative data analysis of images (weights of the oxygen and silver atoms), **(C)** distribution of silver in elemental mapping, and **(D)** shape and size of AgNPs.

For UV–Visible spectroscopy analysis, biogenic AgNPs were dissolved in deionized water and detected using a UV–Visible spectrophotometer (SHIMADZU, UV-1,800). The UV–Visible spectrum showed a strong, broad peak at 400 nm (Figure 3A), and no more major peak shifts were observed during the measurement. As reported by Bu and Lee (2015), the UV spectrum of Ag was found to be 400 nm. UV spectroscopy is an appropriate approach to confirm the formation of AgNPs (Zou et al., 2007), while plant extract showed a peak at 277 nm (Figure 3B). Next, FTIR spectroscopy was performed to identify the chemical groups present in the biogenic AgNPs. The FTIR pattern of AgNPs showed major absorption peaks at 3428.70, 2090.76, 1644.49, and 410.50 cm^–1^ (Figure 3C). The band at 3428.70 cm^–1^ resulted from OH stretching (Vanaja et al., 2013), 2090.76 cm^–1^ attributed to the stretching vibration of hydrocarbon (C–H), which arises from plant metabolites (Thirunavoukkarasu et al., 2013), the band at 1644.49 cm^–1^ is predominant and represents the involvement of the amide-I bond (C = O) of protein as a capping and stabilization agent of silver (Masum et al., 2019), and 410.50 cm^–1^ might have corresponded to SCN bending (Saleh et al., 2016). For a surface charge of AgNPs identification, the sample was appropriately diluted in deionized water to reduce the background. Then, the surface charge (ζ-potential) of the biogenic AgNPs was measured using DLS. The surface charge of biogenic AgNPs has been observed to be −10.8 mV (Figure 3D). ζ-potential measures AgNPs stability by investigating the surface charge potential in aqueous suspensions (Elhawary et al., 2020). A negative charge on the surface of biogenic AgNPs indicates high stability of AgNPs (Römer et al., 2011). Furthermore, the biogenic AgNPs were subjected to EDX analysis. The Oxford EDS instrument was used to detect silver in the nanostructure, elemental mapping, and element distribution of NPs (Figures 4A–C). The quantitative result showed the percentage relative composition of elements such as oxygen (O) at 80% and silver (Ag) at 20% (Figure 4B), and the distribution of AgNPs was homogenous (Figure 4C). The morphological characteristics and particle size of biogenic AgNPs were investigated using SEM. The SEM image demonstrated that the shape of biogenic AgNPs was spherical, with particle sizes ranging from 19 to 26 nm, and the average diameter was found to be 20 nm (Figure 4D). A similar result was reported in the green synthesis of AgNPs using the fruit extract of *Phyllanthus emblica* (Masum et al., 2019). In addition, as reported by Thirunavoukkarasu et al. (2013), most of the AgNPs were spherical.

### The impact of biogenic silver nanoparticles on biomass and antioxidant system

**FIGURE 5:**
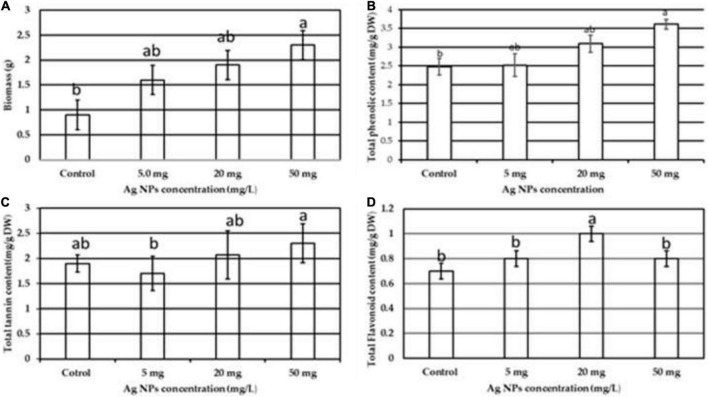
The impact of different doses of biogenic AgNPs on the callus of *J. procera* **(A)** biomass, **(B)** TPC, **(C)** TTC, and **(D)** TFC. ^a,b,c^Means within the same column with different superscripts differ significantly (*P* < 0.05).

**FIGURE 6:**
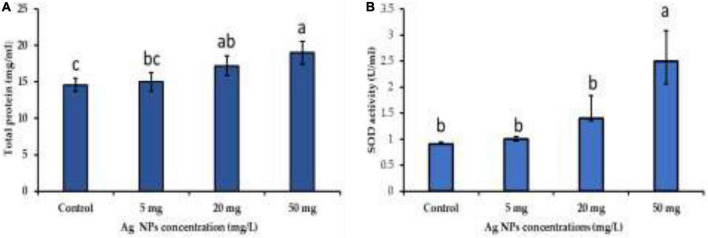
The impact of different doses of biogenic AgNPs on **(A)** total protein content and **(B)** SOD activity. ^a,b,c^Means within the same column with different superscripts differ significantly (*P* < 0.05).

NPs induce several physiological and biochemical reactions in plant cells that might affect plants’ growth positively or negatively, depending on the type, size, concentration, and interaction of NPs with plant cells (Navarro et al., 2008; Khodakovskaya et al., 2012; Thuesombat et al., 2014). In this current work, biogenic AgNPs were employed as elicitors in callus cultures of *J. procera.* The parameters such as biomass and phytochemical constituents were estimated in response to AgNPs treatment. Data in Figure 5 represent the impact of different doses (0.0, 5, 20, and 50 mg/L) of biogenic AgNPs on biomass accumulation and non-enzymatic antioxidants (TPC, TTC, and TFC) production from the callus of *J. procera*. The obtained results demonstrate that biogenic AgNPs significantly impact callus development and phytochemical compounds (TPC, TTC, and TFC) production. In this context, it was reported that AgNPs affect callus growth, proliferation, and secondary metabolites production significantly (Ali et al., 2019). Among different doses, 50 mg/L of AgNPs resulted in the highest biomass accumulation (2.3 g), followed by 20 mg (1.9 g), 5 mg (1.6), and control (0.9 g) (Figure 5A). This may be due to the effect of NPs on physiological and biochemical processes, including metabolism, electron transport chain, and hormone signaling (Paramo et al., 2020). Also, 50 mg of AgNPs recorded the highest value of TPC (3.6 mg/g DW), followed by 20 mg (3.0 mg), 5 mg (2.6 mg/g DW), and control (2.5 mg/g DW) (Figure 5B). Likewise, 50 mg of AgNPs generated the highest value of TTC (2.3 mg/g DW), followed by 20 mg (2.0 mg/d DW), control (1.9 mg/g DW), and 5 mg (1.6 mg/g DW) (Figure 5C). Among different doses, 20 mg of AgNPs achieved the highest yield of TFC (1.0 mg/g DW), followed by 50 mg (0.8 mg/g DW), 5 mg (0.79 mg/g DW), and control (0.7 mg/g DW) (Figure 5D). In general, our findings are in accordance with the recent result reports. For example, a supplement of NPs to the plant media has increased phenolic compound production (Jadczak et al., 2020; Nazir et al., 2021). The increase in phenols and flavonoids production may be due to ROS generation by NPs that starts complicated reactions and affects metabolic processes in the plant cells (Hatami et al., 2019). In this context, there is an indirect relation between secondary metabolites production and ROS. The above findings are supported by physiological investigation, which revealed that 50 mg/L of AgNPs increased total protein content and SOD activity compared to control (Figures 6A,B), respectively. The addition of AgNPs was found to stimulate protein content in the seeds of *Pisum sativum* L. (Mehmood and Murtaza, 2017). Also, the impact of AgNPs on the protein content of *Phaseolus vulgaris* and *Zea mays* was investigated (Salama, 2012), and significant results were recorded. The increase in the enzyme activity might be due to either direct surface interaction of the AgNPs with enzymes or gene regulation (Cameron et al., 2018). On the other hand, no indication or evidence has been observed in this study related to AgNP toxicity.

### The impact of silver nanoparticles on phytochemical compounds

**FIGURE 7:**
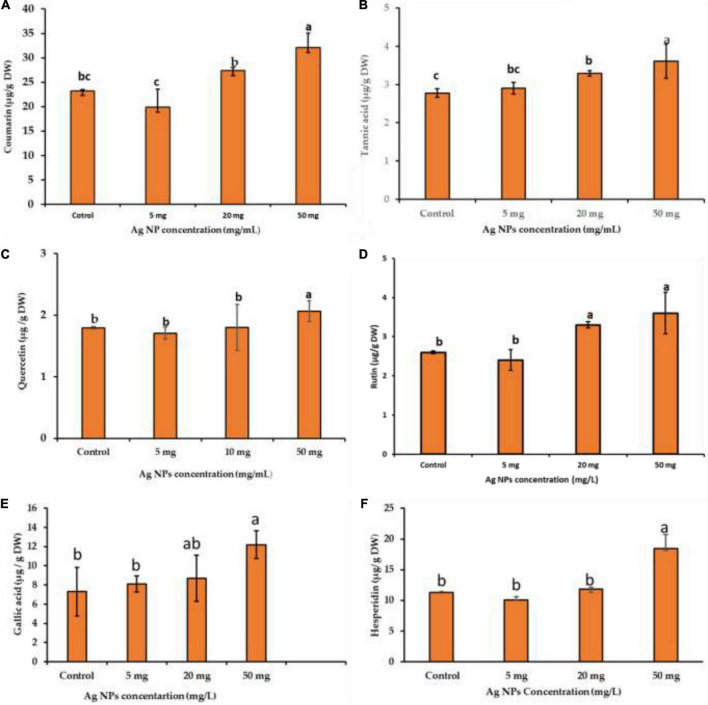
The impact of different concentrations of biogenic AgNPs on bioactive compound production; **(A)** coumarin, **(B)** tannic acid, **(C)** quercetin, **(D)** rutin, **(E)** gallic acid, and **(F)** hesperidin of callus of *J. procera*. ^a,b,c^Means within the same column with different superscripts differ significantly (*P* < 0.05).

Bioactive compounds of medicinal plants are present as natural ingredients which can provide health benefits beyond the basic nutritional value of these products (Biesalski et al., 2009). The availability of some bioactive compounds from current natural sources is limited. Therefore, induction factors are needed to enhance the productivity of phytochemical compounds from medicinal plants for nutritional and pharmaceutical purposes. Using NPs for bioactive component induction is one of the prioritized strategies for the sustainability of bioactive component production (Tian et al., 2018; Vargas-Hernandez et al., 2020; Nazir et al., 2021). Therefore, the impact of biogenic AgNPs on bioactive compounds like coumarin, tannic acid, quercetin, rutin, gallic acid, and hesperidin production from callus was investigated. These compounds were separated and quantified chromatographically using HPLC with reference standards, and specific mobile phases for each compound were used (Figure 1). The obtained results showed that biogenic AgNPs significantly impact the production of bioactive compounds from the callus of *J. procera*. We found that all the investigated constituents, coumarin (Figure 7A), tannic acid (Figure 7B), quercetin (Figure 7C), rutin (Figure 7D), gallic acid (Figure 7E), and hesperidin (Figure 7F), were affected significantly by a higher dose (50 mg/L) of AgNPs. In agreement with our findings, Chung et al. (2018) reported that gallic acid, *p*-coumaric acid, *o*-coumaric acid, quercetin, rutin, and hesperidin were increased significantly in response to AgNPs treatment. In addition, a recent study discovered that CuO and MnO nanomaterials induced phytochemical compounds in the callus of *Ocimum basilicum* (Nazir et al., 2021). The exposure of plants to NPs caused bioactive compound production reported by Marslin et al. (2017). NPs induce a series of physiological and biochemical reactions in the cells of plants and alter phytochemical production (Mulabagal and Tsay, 2004). In addition, there is a relationship between bioactive compounds and ROS (Marslin et al., 2017). For example, compared to the control, treated calluses increased enzymatic antioxidants like SOD and non-enzymatic antioxidants (TPC, TTC, and TFC).

### The effect of biogenic silver nanoparticles on ferruginol production

**FIGURE 8:**
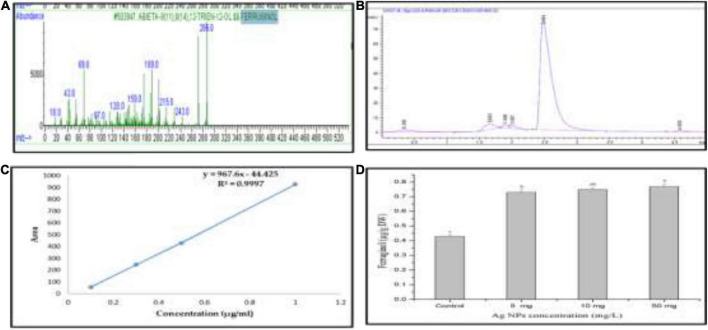
**(A)** Mass spectrum of ferruginol standard curve, **(B)** HPLC chromatogram of ferruginol, **(C)** standard curve of ferruginol, and **(D)** ferruginol production under different doses of AgNPs. ^a,b,c^Means within the same column with different superscripts differ significantly (*P* < 0.05).

Ferruginol, a diterpene phenol, has recently received attention for its pharmacological properties, including antitumor, antimalarial, antibacterial, and cardio-protective effects (Wei et al., 2009; González et al., 2014). Moreover, it has been reported that ferruginol inhibits the growth of cancer cells (González et al., 2014). Recently, we detected ferruginol in the different parts of *J. procera* using GC-MS, DART-MS, and HPLC (Salih et al., 2021a,b; Figure 8A), and it is a dominant compound in different parts of this plant. This study separated ferruginol and identified it using HPLC, with ferruginol standard (Figures 8B,C). For evaluating the effect of biogenic AgNPs on ferruginol production from the callus of *J. procera*, different concentrations (0.0, 5.0, 10, and 50 mg) of AgNPs were used. The achieved results have shown that biogenic AgNPs significantly affect ferruginol production from the callus of *J. procera* (Figure 8D). It has been suggested that nanomaterials interfere with several signaling pathways and are capable of inducing plant secondary metabolite production (Sosan et al., 2016). Also, the exposure of plants to nanomaterials can cause secondary metabolite production (Marslin et al., 2017). Moreover, the increase of secondary metabolites such as ferruginol in response to AgNPs might be due to the regulation of genes.

## Conclusion

In this study, our results demonstrate that biogenic AgNPs significantly impact physico-biochemical processes in the *J. procera* plant. NPs treatments improved callus development and bioactive compound production significantly compared to control. Moreover, this research can serve as a good model for improving the quality of bioactive compounds from medicinal plants *in vitro*. As we know, physiological and biochemical characterizations are not enough. Therefore, a molecular investigation should be conducted to understand better the metabolic process that leads to scaling up the production of bioactive compounds in response to AgNPs treatment.

## Data availability statement

The original contributions presented in this study are included in the article/supplementary material, further inquiries can be directed to the corresponding author.

## Author contributions

AS was responsible for the conceptualization and wrote the original manuscript. AS and SK proposed and planned the research. MN, MT, and HS contributed to the methodology. FA-Q supervised the study. All authors have read and agreed to the published version of the manuscript.

## References

[B1] Abdel Ghany T. Hakamy O. M. (2014). Juniperus procera as food safe additive, their antioxidant, anticancer and antimicrobial activity against some food-borne Bacteria. *J. Biol. Chem. Res.* 31 668–677.

[B2] Adams R. P. (1990). Juniperus procera of East Africa: Volatile leaf oil composition and putative relationship to J. excelsa. *Biochem. Syst. Ecol.* 18 207–210. 10.1016/0305-1978(90)90061-J

[B3] Agnihotri S. Mukherji S. Mukherji S. (2014). Size-controlled silver nanoparticles synthesized over the range 5–100 nm using the same protocol and their antibacterial efficacy. *Rsc Adv.* 4 3974–3983. 10.1039/C3RA44507K

[B4] Ahmad T. Bustam M. A. Irfan M. Moniruzzaman M. Asghar H. M. A. Bhattacharjee S. (2019). Mechanistic investigation of phytochemicals involved in green synthesis of gold nanoparticles using aqueous Elaeis guineensis leaf extracts: Role of phenolic compounds and flavonoids. *Biotechnol. Appl. Biochem.* 66 698–708. 10.1002/bab.1787 31172593

[B5] Ahmed S. Saifullah A. M. Swami B. L. Ikram S. (2016). Green synthesis of silver nanoparticles using Azadirachta indica aqueous leaf extract. *J. Rad. Res. Appl. Sci.* 9 1–7. 10.1016/j.jrras.2015.06.006

[B6] Ainsworth E. A. Gillespie K. M. (2007). Estimation of total phenolic content and other oxidation substrates in plant tissues using Folin–Ciocalteu reagent. *Nat. Protocols* 2 875–877. 10.1038/nprot.2007.102 17446889

[B7] Ali A. Mohammad S. Khan M. A. Raja N. I. Arif M. Kamil A. (2019). Silver nanoparticles elicited in vitro callus cultures for accumulation of biomass and secondary metabolites in Caralluma tuberculata. *Artif. Cells Nanomed, Biotechnol.* 47 715–724. 10.1080/21691401.2019.1577884 30856344

[B8] Ashraf J. M. Ansari M. A. Khan H. M. Alzohairy M. A. Choi I. (2016). Green synthesis of silver nanoparticles and characterization of their inhibitory effects on AGEs formation using biophysical techniques. *Sci. Rep.* 6:20414. 10.1038/srep20414 26829907 PMC4735866

[B9] Banerjee P. Satapathy M. Mukhopahayay A. Das P. (2014). Leaf extract mediated green synthesis of silver nanoparticles from widely available Indian plants: synthesis, characterization, antimicrobial property and toxicity analysis. *Bioresour. Bioproc.* 1:3. 10.1186/s40643-014-0003-y

[B10] Biesalski H.-K. Dragsted L. O. Elmadfa I. Grossklaus R. Müller M. Schrenk D. (2009). Bioactive compounds: Definition and assessment of activity. *Nutrition* 25 1202–1205. 10.1016/j.nut.2009.04.023 19695833

[B11] Bitew D. (2015). Assessment of the inhibitory activity of resin from Juniperus procera against the mycilium of Pyrofomes demidoffi. *J. Plant Pathol. Microb.* 6:2.

[B12] Bu Y. Lee S. W. (2015). The characteristic Ag(core)Au(shell) nanoparticles as SERS substrates in detecting dopamine molecules at various pH ranges. *Int. J. Nanomed.* 10 Spec Iss 47–54. 10.2147/IJN.S88308 26345418 PMC4554420

[B13] Cameron S. J. Hosseinian F. Willmore W. G. (2018). A current overview of the biological and cellular effects of nanosilver. *Int. J. Mol. Sci.* 19:2030. 10.3390/ijms19072030 30002330 PMC6073671

[B14] Chandran S. P. Chaudhary M. Pasricha R. Ahmad A. Sastry M. (2006). Synthesis of gold nanotriangles and silver nanoparticles using Aloevera plant extract. *Biotechnol. Prog.* 22 577–583. 10.1021/bp0501423 16599579

[B15] Chung I.-M. Rajakumar G. Thiruvengadam M. (2018). Effect of silver nanoparticles on phenolic compounds production and biological activities in hairy root cultures of Cucumis anguria. *Acta. Biol. Hungarica* 69 97–109. 10.1556/018.68.2018.1.8 29575919

[B16] Collenette S. (1999). *Wild flowers of Saudi Arabia*, Vol. 110. Norwich: East Anglian Engraving Co. Ltd, 274–275.

[B17] Durán N. Silveira C. P. Durán M. Martinez D. S. T. (2015). Silver nanoparticle protein corona and toxicity: a mini-review. *J. Nanobiotechnol.* 13 1–17. 10.1186/s12951-015-0114-4 26337542 PMC4559354

[B18] Elhawary S. Hala E.-H. Mokhtar F. A. Mansour Sobeh E. M. Osman S. El-Raey M. (2020). Green synthesis of silver nanoparticles using extract of Jasminum officinal l. leaves and evaluation of cytotoxic activity towards bladder (5637) and breast cancer (MCF-7) cell lines. *Int. J. Nanomed.* 15:9771. 10.2147/IJN.S269880 33304101 PMC7723236

[B19] Frewer L. J. Gupta N. George S. Fischer A. Giles E. L. Coles D. (2014). Consumer attitudes towards nanotechnologies applied to food production. *Trends Food Sci. Technol.* 40 211–225. 10.1007/s11051-015-3270-4 26660049 PMC4666279

[B20] García-Sánchez S. Bernales I. Cristobal S. (2015). Early response to nanoparticles in the Arabidopsis transcriptome compromises plant defence and root-hair development through salicylic acid signalling. *BMC Genomics* 16:341. 10.1186/s12864-015-1530-4 25903678 PMC4417227

[B21] González M. A. Clark J. Connelly M. Rivas F. (2014). Antimalarial activity of abietane ferruginol analogues possessing a phthalimide group. *Bioorganic Med. Chem. Lett.* 24 5234–5237. 10.1016/j.bmcl.2014.09.061 25316317

[B22] Hatami M. Naghdi Badi H. Ghorbanpour M. (2019). Nano-elicitation of secondary pharmaceutical metabolites in plant cells: A review. *J. Med. Plants* 18 6–36. 10.29252/jmp.3.71.6

[B23] Hochella M. F. Lower S. K. Maurice P. A. Penn R. L. Sahai N. Sparks D. L. (2008). Nanominerals, mineral nanoparticles, and earth systems. *Science* 319 1631–1635. 10.1126/science.1141134 18356515

[B24] Iravani S. (2011). Green synthesis of metal nanoparticles using plants. *Green Chem.* 13 2638–2650. 10.1039/c1gc15386b

[B25] Iravani S. Korbekandi H. Mirmohammadi S. V. Zolfaghari B. (2014). Synthesis of silver nanoparticles: chemical, physical and biological methods. *Res. Pharm. Sci.* 9:385. 26339255 PMC4326978

[B26] Jadczak P. Kulpa D. Drozd R. Przewodowski W. Przewodowska A. (2020). Effect of AuNPs and AgNPs on the Antioxidant System and Antioxidant Activity of Lavender (Lavandula angustifolia Mill.) from In Vitro Cultures. *Molecules* 25:5511. 10.3390/molecules25235511 33255548 PMC7728155

[B27] Jeevanandam J. Barhoum A. Chan Y. S. Dufresne A. Danquah M. K. (2018). Review on nanoparticles and nanostructured materials: history, sources, toxicity and regulations. *Beilstein J. Nanotechnol.* 9 1050–1074. 10.3762/bjnano.9.98 29719757 PMC5905289

[B28] Jogeswar G. Pallela R. Jakka N. Reddy P. Rao J. V. Sreenivasulu N. (2006). Antioxidative response in different sorghum species under short-term salinity stress. *Acta. Physiol. Plantarum* 28 465–475. 10.1007/BF02706630

[B29] Khalil M. M. Ismail E. H. El-Baghdady K. Z. Mohamed D. (2014). Green synthesis of silver nanoparticles using olive leaf extract and its antibacterial activity. *Arabian J. Chem.* 7 1131–1139. 10.3390/ijms222212562 34830442 PMC8621457

[B30] Khodakovskaya M. V. De Silva K. Biris A. S. Dervishi E. Villagarcia H. (2012). Carbon nanotubes induce growth enhancement of tobacco cells. *ACS Nano* 6 2128–2135. 10.1021/nn204643g 22360840

[B31] Kumar P. Selvi S. S. Govindaraju M. (2013). Seaweed-mediated biosynthesis of silver nanoparticles using Gracilaria corticata for its antifungal activity against Candida spp. *Appl. Nanosci.* 3 495–500. 10.1007/s13204-012-0151-3

[B32] Marklund S. Marklund G. (1974). Involvement of the superoxide anion radical in the autoxidation of pyrogallol and a convenient assay for superoxide dismutase. *Eur. J. Biochem.* 47 469–474. 10.1111/j.1432-1033.1974.tb03714.x 4215654

[B33] Marslin G. Sheeba C. J. Franklin G. (2017). Nanoparticles alter secondary metabolism in plants via ROS burst. *Front. Plant Sci.* 8:832. 10.3389/fpls.2017.00832 28580002 PMC5437210

[B34] Masum M. Islam M. Siddiqa M. Ali K. A. Zhang Y. Abdallah Y. (2019). Biogenic synthesis of silver nanoparticles using Phyllanthus emblica fruit extract and its inhibitory action against the pathogen Acidovorax oryzae strain RS-2 of rice bacterial brown stripe. *Front. Microbiol.* 10:820. 10.3389/fmicb.2019.00820 31110495 PMC6501729

[B35] Mehmood A. Murtaza G. (2017). Impact of biosynthesized silver nanoparticles on protein and carbohydrate contents in seeds of Pisum sativum L. *Crop Breed. Appl. Biotechnol.* 17 334–340. 10.1590/1984-70332017v17n4a51

[B36] Mirzajani F. Askari H. Hamzelou S. Schober Y. Römpp A. Ghassempour A. (2014). Proteomics study of silver nanoparticles toxicity on Oryza sativa L. *Ecotoxicol. Environ. Safe.* 108 335–339. 10.1016/j.ecoenv.2014.07.013 25124680

[B37] Mohanpuria P. Rana N. K. Yadav S. K. (2008). Biosynthesis of nanoparticles: technological concepts and future applications. *J. Nanoparticle Res.* 10 507–517. 10.1007/s11051-007-9275-x

[B38] Mulabagal V. Tsay H.-S. (2004). Plant cell cultures-an alternative and efficient source for the production of biologically important secondary metabolites. *Int. J. Appl. Sci. Eng.* 2 29–48.

[B39] Muley B. Khadabadi S. Banarase N. (2009). Phytochemical constituents and pharmacological activities of Calendula officinalis Linn (Asteraceae): a review. *Tropical. J. Pharm. Res.* 8 455–465. 10.4314/tjpr.v8i5.48090

[B40] Navarro E. Baun A. Behra R. Hartmann N. B. Filser J. Miao A.-J. (2008). Environmental behavior and ecotoxicity of engineered nanoparticles to algae, plants, and fungi. *Ecotoxicology* 17 372–386. 10.1007/s10646-008-0214-0 18461442

[B41] Nazir S. Jan H. Zaman G. Khan T. Ashraf H. Meer B. (2021). Copper oxide (CuO) and manganese oxide (MnO) nanoparticles induced biomass accumulation, antioxidants biosynthesis and abiotic elicitation of bioactive compounds in callus cultures of Ocimum basilicum (Thai basil). *Artif. Cells Nanomed. Biotechnol.* 49 626–634. 10.1080/21691401.2021.1984935 34597252

[B42] Nour V. Trandafir I. Cosmulescu S. (2013). HPLC determination of phenolic acids, flavonoids and juglone in walnut leaves. *J. Chromatogr. Sci.* 51 883–890. 10.1093/chromsci/bms180 23135132

[B43] Ordonez A. Gomez J. Vattuone M. (2006). Antioxidant activities of Sechium edule (Jacq.) Swartz extracts. *Food Chem.* 97 452–458. 10.1016/j.foodchem.2005.05.024

[B44] Ovais M. Khalil A. T. Islam N. U. Ahmad I. Ayaz M. Saravanan M. (2018). Role of plant phytochemicals and microbial enzymes in biosynthesis of metallic nanoparticles. *Appl. Microbiol. Biotechnol.* 102 6799–6814. 10.1007/s00253-018-9146-7 29882162

[B45] Paramo L. A. Feregrino-Pérez A. A. Guevara R. Mendoza S. Esquivel K. (2020). Nanoparticles in agroindustry: Applications, toxicity, challenges, and trends. *Nanomaterials* 10:1654. 10.3390/nano10091654 32842495 PMC7558820

[B46] Rauwel P. Küünal S. Ferdov S. Rauwel E. (2015). A review on the green synthesis of silver nanoparticles and their morphologies studied via TEM. *Adv. Mater. Sci. Eng.* 2015:682749. 10.1155/2015/682749

[B47] Rodrigues C. I. Marta L. Maia R. Miranda M. Ribeirinho M. Máguas C. (2007). Application of solid-phase extraction to brewed coffee caffeine and organic acid determination by UV/HPLC. *J. Food Compos. Anal.* 20 440–448. 10.1016/j.jfca.2006.08.005

[B48] Römer I. White T. A. Baalousha M. Chipman K. Viant M. R. Lead J. R. (2011). Aggregation and dispersion of silver nanoparticles in exposure media for aquatic toxicity tests. *J. Chromatogr. A* 1218 4226–4233. 10.1016/j.chroma.2011.03.034 21529813

[B49] Salama H. M. (2012). Effects of silver nanoparticles in some crop plants, common bean (Phaseolus vulgaris L.) and corn (Zea mays L.). *Int. Res. J. Biotechnol.* 3 190–197.

[B50] Saleh T. A. Al-Shalalfeh M. M. Al-Saadi A. A. (2016). Graphene Dendrimer-stabilized silver nanoparticles for detection of methimazole using Surface-enhanced Raman scattering with computational assignment. *Sci. Rep.* 6:32185. 10.1038/srep32185 27572919 PMC5004140

[B51] Salih A. M. Al-Qurainy F. Khan S. Tarroum M. Nadeem M. Shaikhaldein H. O. (2021a). Mass propagation of Juniperus procera Hoechst. Ex Endl. From seedling and screening of bioactive compounds in shoot and callus extract. *BMC Plant Biol.* 21:192. 10.1186/s12870-021-02946-2 33882830 PMC8059214

[B52] Salih A. M. Al-Qurainy F. Khan S. Tarroum M. Nadeem M. Shaikhaldein H. O. (2021b). Biosynthesis of zinc oxide nanoparticles using Phoenix dactylifera and their effect on biomass and phytochemical compounds in Juniperus procera. *Sci. Rep.* 11:19136. 10.1038/s41598-021-98607-3 34580362 PMC8476557

[B53] Sosan A. Svistunenko D. Straltsova D. Tsiurkina K. Smolich I. Lawson T. (2016). Engineered silver nanoparticles are sensed at the plasma membrane and dramatically modify the physiology of Arabidopsis thaliana plants. *Plant J.* 85 245–257. 10.1111/tpj.13105 26676841

[B54] Srikar S. K. Giri D. D. Pal D. B. Mishra P. K. Upadhyay S. N. (2016). Green synthesis of silver nanoparticles: a review. *Green Sustain. Chem.* 6 34–56.

[B55] Suleiman R. K. Iali W. El Ali B. Umoren S. A. (2021). New Constituents from the Leaves of Date Palm (Phoenix dactylifera L.) of Saudi Origin. *Molecules* 26:4192. 10.3390/molecules26144192 34299467 PMC8306910

[B56] Syedmoradi L. Daneshpour M. Alvandipour M. Gomez F. A. Hajghassem H. Omidfar K. (2017). Point of care testing: The impact of nanotechnology. *Biosens. Bioelectron.* 87 373–387. 27589400 10.1016/j.bios.2016.08.084

[B57] Thirunavoukkarasu M. Balaji U. Behera S. Panda P. Mishra B. (2013). Biosynthesis of silver nanoparticle from leaf extract of Desmodium gangeticum (L.) DC. and its biomedical potential. *Spectrochim. Acta. Part A* 116 424–427. 10.1016/j.saa.2013.07.033 23973589

[B58] Thuesombat P. Hannongbua S. Akasit S. Chadchawan S. (2014). Effect of silver nanoparticles on rice (Oryza sativa L. cv. KDML 105) seed germination and seedling growth. *Ecotoxicol. Environ. Safe.* 104 302–309. 10.1016/j.ecoenv.2014.03.022 24726943

[B59] Tian H. Ghorbanpour M. Kariman K. (2018). Manganese oxide nanoparticle-induced changes in growth, redox reactions and elicitation of antioxidant metabolites in deadly nightshade (Atropa belladonna L.). *Industrial Crops Products* 126 403–414.

[B60] Tumen I. Eller F. J. Clausen C. A. Teel J. A. (2013). Antifungal activity of heartwood extracts from three Juniperus species. *BioResources* 8 12–20.

[B61] Vanaja M. Gnanajobitha G. Paulkumar K. Rajeshkumar S. Malarkodi C. Annadurai G. (2013). Phytosynthesis of silver nanoparticles by Cissus quadrangularis: influence of physicochemical factors. *J. Nanostruct. Chem.* 3:17.

[B62] Vargas-Hernandez M. Macias-Bobadilla I. Guevara-Gonzalez R. G. Rico-Garcia E. Ocampo-Velazquez R. V. Avila-Juarez L. (2020). Nanoparticles as potential antivirals in agriculture. *Agriculture* 10:444. 10.3390/agriculture10100444

[B63] Wei Y. He J. Qin H. Wu X. A. Yao X. (2009). Determination of ferruginol in rat plasma via high-performance liquid chromatography and its application in pharmacokinetics study. *Biomed. Chromatogr.* 23 1116–1120. 10.1002/bmc.1232 19444795

[B64] Zou J. Xu Y. Hou B. Wu D. Sun Y. (2007). Controlled growth of silver nanoparticles in a hydrothermal process. *China Particuol.* 5 206–212. 10.1016/j.cpart.2007.03.006

